# 1625. Challenges in Addressing Congenital Syphilis in a Large Hospital System

**DOI:** 10.1093/ofid/ofad500.1460

**Published:** 2023-11-27

**Authors:** Gilhen Rodriguez, Sebastian Shrager, Stacy Gomez Hernandez, Gloria Heresi, Asvini Thiviyanathan, James Murphy, Norma Perez

**Affiliations:** University of Texas McGovern Medical School, Houston, Texas; UT Health Science Center at Houston, Houston, Texas; UTHealth, McGovern Medical School, Houston, Texas; UTHealth, McGovern Medical School, Houston, Texas; UT Health Science Center at Houston, Houston, Texas; UTHealth, McGovern Medical School, Houston, Texas; University of Texas McGovern Medical School, Houston, Texas

## Abstract

**Background:**

Congenital Syphilis (CS) has increased markedly in the Houston-TX metropolitan region; the rate per 100,000 live births was 138.6 in 2018. Therefore, we reviewed CS exposures presented to our network of institutions to identify possible changes in practice having the potential to improve CS management.

**Table 1.**
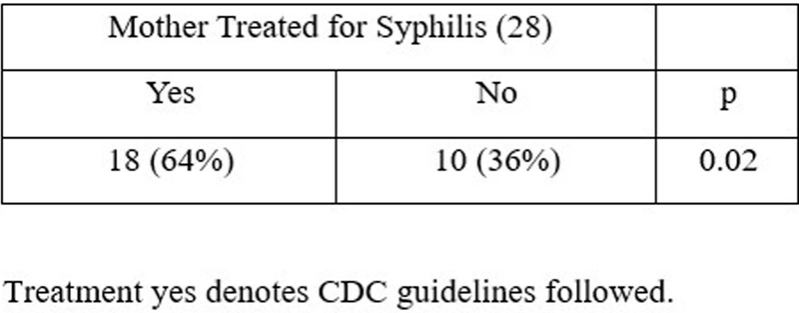
RPR-Reactive Newborns of Mothers Receiving Prenatal Care.

**Methods:**

Retrospective record review. From June 2015 through July 2022, 754 RPR+ at-delivery mothers were identified. A random sample of 25% was selected for an ongoing investigation. Eighty-seven mothers and their 89 newborns have had their medical records extracted and analyzed for the presented analysis. Prenatal care was defined as ≥ 3 visits to an obstetrics provider. Maternal treatment of syphilis was categorized as complete when the CDC criteria were met. The χ^2^ test was used, and significance was set to *P* ≤ 0.05. The institutional IRB approved the study.

**Results:**

Notable characteristics of the 87 mothers were 68% African American, 81% not Hispanic, 79% single parents with median gestation of 3 (1-11) and age of 26 (±6).

Of the 89 newborns, 54% were female, 57% were full-term, and 51% were delivered by C-section. In addition, 53% were RPR-reactive at birth, and 57% were treated for CS.

Analyses targeting interventions doable within our institutional circumstances revealed that 36% of mothers who received prenatal care and delivered newborns who were RPR-reactive at birth had no record of receiving recommended treatment for syphilis, Table1.

**Conclusion:**

Our population has a well-established pattern of risk factors for CS, including the use of illicit drugs. In addition, our medical record review identified a possible failure to provide or record treatment for syphilis for women who received prenatal care and delivered RPR-reactive infants. Therefore, an effort is being directed to improve these outcomes.

**Disclosures:**

**All Authors**: No reported disclosures

